# Moving policy to practice: role of advocacy in enabling provision of injectable contraceptives by pharmacists in Kenya

**DOI:** 10.3389/fgwh.2023.1218220

**Published:** 2023-10-11

**Authors:** Sally Njiri, Sam Mulyanga, Irene Choge, Beatrice Kwachi, Rammah Mwalimu, Susan Ontiri

**Affiliations:** ^1^ Advance Family Planning Project, Jhpiego, Nairobi, Kenya; ^2^ International Center for Reproductive Health, Mombasa, Kenya

**Keywords:** family planning, pharmacist, advocacy, Kenya, policy

## Abstract

Expanding access to contraceptive services by making them available in pharmacies and drug shops is a family planning high-impact practice. In 2018, Kenya's Ministry of Health amended its family planning guidelines to allow pharmacists and pharmaceutical technologists throughout the country to provide subcutaneous and intramuscular depot medroxyprogesterone acetate. Amending the policy did not necessarily mean that the policy would be implemented. The Advance Family Planning project launched an advocacy campaign to engage key stakeholders to work with the Ministry of Health to implement the policy. Consequently, a family planning training package for pharmacists and pharmaceutical technologists was developed and rolled out. The advocacy process also led to strengthening family planning reporting by the trained pharmacists and pharmaceutical technologists. To further enhance sustainability by ensuring a continuous pool of pharmacy professionals equipped with skills to provide family planning services, Advance Family Planning and its partners advocated with universities and the Pharmacy and Poisons Board to revise the pre-service training curriculum to include family planning as a competence area for pharmacists and pharmaceutical technologists. A key lesson learned is that policy formulation does not necessarily translate to policy implementation. Advocacy is often needed to move policy to practice, especially where resources are required. Policy implementation also requires incremental achievement of milestones and the need for advocacy for each step in the process. Implementation of the policy provision that allows pharmacists and pharmaceutical technologists to provide injectable contraceptives has implications beyond family planning programs. It provides a point of reference for allowing pharmacists to offer other primary health care services, such as immunization, injectable HIV prophylaxis, and other interventions that might not be provided for in policy.

## 1. Introduction

Expanding access to contraceptive services using pharmacies and drug shops is one of the high-impact practices in family planning (FP) service delivery (1). In low- and middle-income countries, such as those in sub-Saharan Africa, pharmacies and drug shops are usually the first point of contact for health care services (2). This is because they are more accessible than health facilities due to their neighborhood location; hence, they can more easily serve even marginalized populations, such as those residing in poor and rural areas. Their flexible operating hours—late into the night, including over the weekend—makes them easily accessible, including by adolescents and youths (1, 3).

Several studies have been documented to show the feasibility of provision of contraceptive services through pharmacies and drug shops. Currently, many users of short-term contraceptive methods, specifically contraceptive pills and condoms, access them through pharmacies and drug shops.(1) In Nigeria, a study revealed that younger respondents, single people, Catholics, and Muslims preferred to obtain contraceptive method and information from drug shops than other sources (4, 5). In Kenya, almost half (45%) women who use pills and 16% of people who use condoms access their method through pharmacies (6). As such, there has been a call to expand the methods that pharmacies can provide beyond pills and condoms (2). The World Health Organization's 2017 FP task-sharing guideline recommended the expansion of contraceptives services provided by pharmacists to include injectable contraceptives (7). This is critical considering that there are an inadequate number of health workers and injectable contraceptives continue to be the most popular method in sub-Saharan Africa; increasing its accessibility beyond health facilities to pharmacies and drug shops could potentially improve continuation of injectable contraceptives (5, 8).

Following the World Health Organization's guidance, many countries, including Kenya, embraced task sharing and shifting in FP program to allow pharmacists and drug shops to offer injectable contraceptives. In 2018, Kenya's Ministry of Health (MOH) amended its FP guidelines for service providers to allow pharmacists and pharmaceutical technologists (P&PT) throughout the country to provide subcutaneous and intramuscular depot medroxyprogesterone acetate (DMPA-SC and DMPA-IM) (7). Despite this policy being in place, certain barriers hindered its implementation. First, the country lacked guidelines on how this policy can be implemented; FP is not usually included in pre-service training for pharmacists. Moreover, P&PT, who mostly work in the private sector, do not get the opportunity to take part in in-service trainings organized by the MOH due to concerns such as the inability to close facilities to attend a week-long training (8). Second, programmatic reports indicate that the Ministry of Health was concerned about the quality of care provided in pharmacies. There have been concerns around components related to counselling and referral as most pharmacies lack adequate infrastructure that can guarantee auditory privacy for women seeking contraceptive services who might need counselling. In addition, most pharmacists work in the private sector and do not have a direct link with the MOH, specifically the FP program that ensures all FP providers offer the expected standard of care. Third, pharmacists don't routinely report in the national health management information system, making it difficult to track the services they offer.

Aware that translating policies into practice can be a challenge, the Advance Family Planning (AFP) project, which focused on policy and budget advocacy, spearheaded measures to operationalize the policy to allow pharmacists and drug shops to offer injectable contraceptives and to ensure that the policy is implemented as was envisaged. This article outlines the process that AFP, in collaboration with pharmaceutical associations and other stakeholders, took to advocate for the policy to be implemented. In this article, we document our advocacy experience in implementation of the national guidelines that allowed P&PT to provide injectable contraceptives that led to the development of training packages and inclusion of the module in the preservice training curriculum. We believe this process can be applied to other sectors and other countries that are struggling to translate policy into action.

## 2. Implementation

### 2.1. Advocacy for implementation of the national guidelines

Prior to 2018, the Kenya Family Planning Guidelines for Service Providers only allowed P&PT to provide condoms and contraceptive pills. For more than three years, AFP's local partner, Jhpiego, worked with the Pharmaceutical Society of Kenya and other key stakeholders to successfully advocate to have the guidelines amended to allow P&PT to provide injectable contraceptives. Consequently, Kenya's 2018 National Family Planning Guidelines for Service Providers (6th edition) recognizes that FP trained pharmacists and pharmaceutical technologists can counsel and provide injectable contraceptives in addition to pills and condoms and refer for long acting and permanent methods.

**Figure 1:**
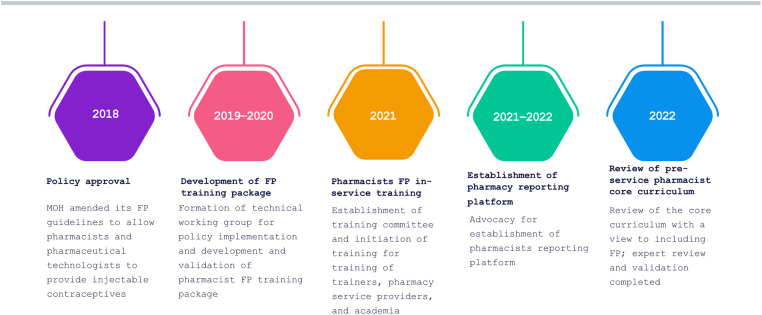
Timeline for moving policy to implementation.

Despite the 2018 policy amendment, a win that assured an enabling policy environment, the policy lacked an implementation plan. Advocacy was needed to translate the new amendment into practice (Figure 1). For instance, P&PT could not provide injectable contraceptive services to clients without being trained, but the MOH had not developed a training package. AFP worked with stakeholders to advocate for specific objectives that led to the implementation of the new policy provision, starting with development of the FP training package for P&PT. We used the SMART Advocacy (specific, measurable, attainable, relevant and time-bound) approach to engage with decision-makers in the MOH's Department of Family Health and Division of Reproductive and Maternal Health who had the power to authorize implementation of the policy provision.

### 2.2. Advocacy for development of training package

AFP successfully advocated for the MOH to develop a FP training package for P&PT by making a case to the head of the Division of Reproductive and Maternal Health on the rationale and need to establish a technical committee that would lead the drafting of the training package. Subsequently, in April 2019, the MOH, through the Division of Reproductive Health and Maternal Health, established a technical committee with a clear mandate to lead the development of the training package under the oversight of the division. The committee included members from the Division of Reproductive and Maternal Health; the Pharmacy and Poisons Board (PPB), the regulatory body for drugs and the pharmacy practice; Kenya Pharmaceutical Association (KPA), which represents pharmaceutical technologists; Pharmaceutical Society of Kenya (PSK), which represents pharmacists; and key FP implementing partners.

The committee developed a draft training package that includes the trainers' manual, which guides the facilitators in conducting the training; the participants' manual, which contains detailed reference information for trainees; and the logbook, in which a trainee logs all procedures they will need for certification. The training package was pretested and validated by key stakeholders in December 2019. Core elements of the training package included anatomy and physiology, counselling, medical eligibility criteria, contraceptive methods, and infection prevention and control. It also covered integration and referrals, reporting, commodity management, and pharmacovigilance. The package clearly outlines issues of accreditation for pharmacy outlets and certification of trainees.

There was a pause in activity following the transition of the Head of the Department of Family Health. In June 2020, AFP partner Jhpiego mobilized other stakeholders inside and outside the MOH to brief the new head; he signed off on the training package a month later.

### 2.3. Advocacy for initiation of in-service training

**Figure 2:**
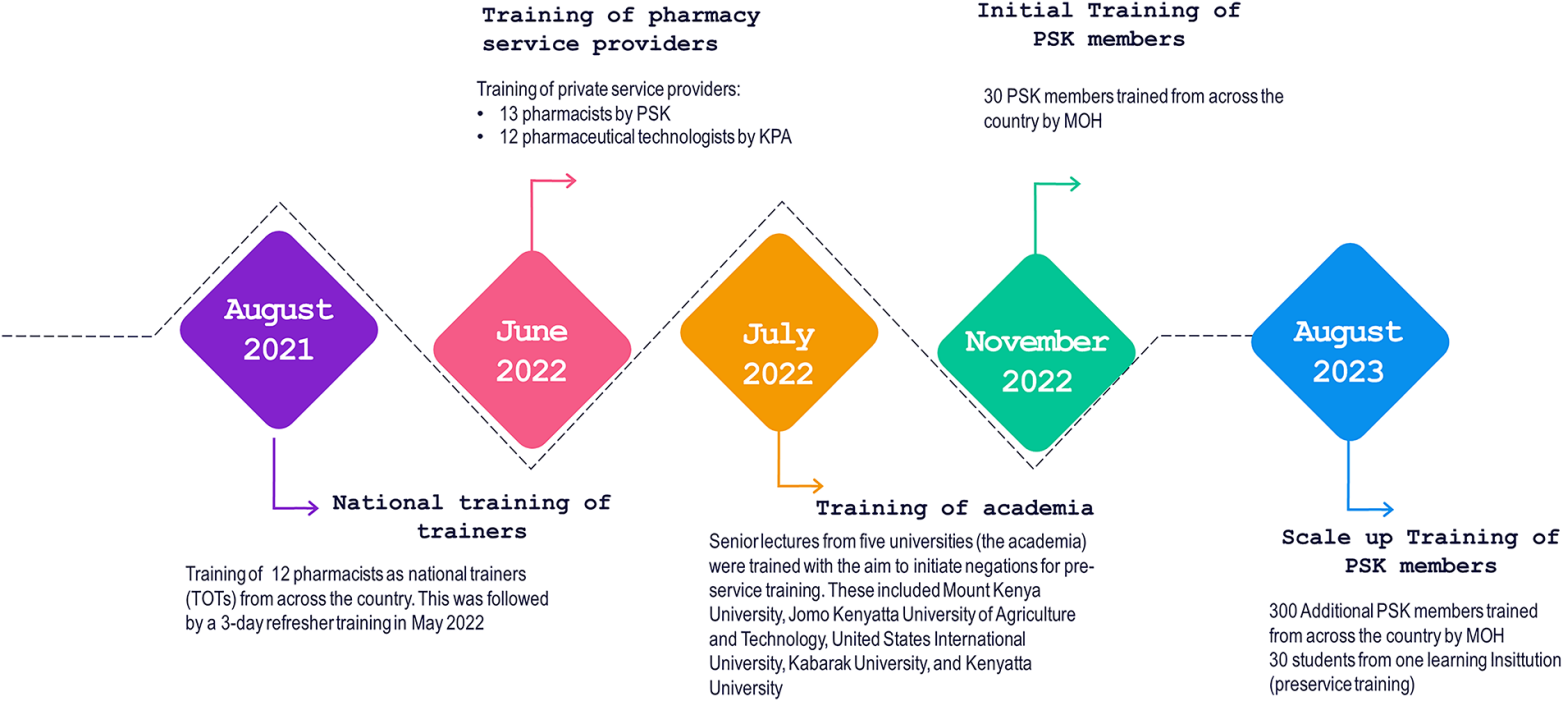
Cascading family planning in-service training of pharmacists in Kenya.

Following the development of the training package, the technical manager in-charge of FP at the MOH engaged County Directors of Health and the County Reproductive Health Coordinators to get their buy-in to roll out the training. This was essential because following the training, the pharmacists and pharmaceutical technologists were expected to report to the counties. The national training coordination committee spearheaded the training of master trainers, the heads of pharmacy departments in higher (Universities)—and tertiary institutions such as Kenya Medical Training Colleges, and service providers from community pharmacies. A team led by PSK, KPA, and the PPB selected and identified training participants who were selected from Kenya's eight geographical regions (Eastern, Nairobi, Central, Coast, Northeastern, Nyanza, Rift Valley, and Western). The team also worked to balance the composition between pharmaceutical technologists and pharmacists. Participants were required to be registered and enrolled by PPB and to have expressed an interest in the training, which ensured that the program was demand driven and not a push system and created ownership among the interested pharmacists. Figure 2 provides details of the teams trained.

### 2.4. Cascading pharmacy Fp training in Kenya

The national MOH and the counties' Department of Health took the central lead in coordinating training, specifically the county Reproductive Health Coordinator and the County Pharmacist in counties where practicums were held. Health facilities with high numbers of FP consumers were selected to be practicum sites. The training covered all aspects in the training package and included theory sessions, practicums for skills transfer through use of humanistic models, and skills training in the health facilities. The MOH provided FP commodities for the practicum.

Following the completion of the five-day in-person FP training, which involved three days of theory and two days of clinical skill practicum followed by clinical assessment, the trainees were awarded a certificate of participation. Both the certificate and logbook are co-signed by the MOH and the PSK and the KPA.

### 2.5. Advocacy for establishment of national pharmacy reporting platform

Until recently, private pharmacies were not reporting in the national health information reporting system the FP services they provided. While the PPB Act requires P&PT to report to the national health management information system, compliance was weak. As a result, the MOH did not have comprehensive data to inform decision-making, especially in areas such as commodity forecasting and quantification.

Following advocacy by AFP, the MOH established a reporting subcommittee within the broader FP pharmacy training coordination committee that included staff from the FP program within Division of Reproductive and Maternal Health, PSK members, and AFP staff. The subcommittee advocated for the establishment of a pharmacy reporting platform within the national health information with the leadership of the MOH Department of Monitoring, Evaluation, and Informatics and the PPB. As a result, the Department of Monitoring, Evaluation and Informatics developed a pharmacy reporting platform prototype through a co-creation approach with stakeholders. The prototype used unique identifiers for registered and licensed pharmacists supplied by the PPB. The department aligned the prototype with health facility FP reporting tools. The pharmacy reporting platform was further enhanced, pretested, and validated by stakeholders.

Upon finalization of the pharmacy reporting platform, the Director General of the MOH issued a circular to all counties in Kenya describing the system and next steps. The Department of Monitoring, Evaluation and Informatics took advantage of the FP P&PT training to conduct orientation on the new reporting platform. Follow-on support was also provided to the trainees by the department post-orientation. Regular progress review meetings that brought trainees together also provided an opportunity to assess progress, troubleshoot reporting challenges, interact with the reporting system, and offer real-time system support. According to the reporting platform about 40 percent were reporting on the pharmacy platform as of December 2022.

### 2.6. Advocacy for pre-service training in higher-education institutions

To sustain FP training and enable a continuous pool of pharmacy professionals equipped with skills to provide FP services, it was imperative that training be included in the PPB's pre-service core curriculum that provides a minimum package for training for P&PT. Universities and middle-level colleges in Kenya draw from this curriculum when developing their own curricula.

AFP, the MOH Department for Family Health, and PSK engaged with the Training Technical Committee of the PPB to advocate for review of the core curriculum with a view to including FP. AFP had earlier conducted a landscape assessment that showed that the core curriculum was last revised in 2014, and was due for review in 2019, hence the time was ripe for a revision. In addition, there was a growing concern within the pharmacy profession for the need to change the perception of pharmacists from simply dispensers of drugs to providers of care. There was also an emerging appreciation of the need to make education more practical and less theoretical. Following the advocacy efforts, the Training Technical Committee and the Chief Executive Officer of the PPB sanctioned the revision of the core curriculum.

The Training Technical Committee embarked on the revision of the core curriculum for both universities and middle-level colleges, which was reviewed by external experts drawn mainly from education institutions. Once input from the experts had been incorporated in the curricula, the draft curricula was then subjected to validation. The curriculum was validated by key stakeholders, including the Kenya Qualification Authority, Commission for University Education, technical and vocational training regulatory bodies, individual universities and colleges offering pharmacy, and professional bodies, specifically PSK and KPA.

The validated core curriculum included new elements such as FP, immunization, supply chain, gender and health, and governance and leadership, among others. The curriculum shifted towards practicums and care aspects of the pharmacy practice to align it to emerging needs and current realities.

AFP worked through PSK to advocate for implementation of pharmacists' FP training module within the pharmacists' training curriculum as a pilot within institutions of higher learning. A key aspect of this training focused on injectable contraceptives, including the associated counselling and clinical skills. Five public and private universities engaged in the pre-service training conversations: Mount Kenya University, Jomo Kenyatta University of Agriculture and Technology, United States International University, Kabarak University, and Kenyatta University. To enhance the prospect of incorporating FP in individual university curricula and training, AFP, working with the MOH and PSK, conducted training using the MOH in-service FP training package. The trainees were drawn from deans and lecturers in the pharmacy departments of the five participating institutions; each institution developed an implementation action plan for FP training.

Regular quarterly reviews were conducted to assess progress on the action plans' implementation. By the end of 2022, one university had amended its curriculum to include FP, four universities had included FP in their student rotation/attachment logbooks, and one university had trained 130 of its students in FP, including clinical skills on how to provide injectable contraceptives. It is envisaged that the pre-service core curriculum by the PPB will spur additional universities and colleges to implement FP training in their respective schools of pharmacy.

### 2.7. Advocacy for quality assurance

The MOH's national FP program is mandated to ensure quality of FP services and build the capacity of and provide technical assistance to counties in the delivery of quality FP services. To ensure quality of training and FP service provision, the following were considered:

• **Certification:** Once a P&PT trainee has successfully completed the training, they will be awarded a certificate of participation by the MOH and the PSK or KPA. A health facility and a preceptor/mentor will be identified to supervise recording of completed procedures in a trainee's logbook, which they receive at the start of training to track the number of procedures needed to acquire competency. After trainees complete the number of recommended procedures, they submit their logbook to the MOH for verification. Trainees will then be awarded a certificate of competency co-signed by the MOH and the PSK or KPA.
• **Eligibility Criteria**: The target group for this course is P&PT who will be involved in providing FP services. Selection of P&PT who will be FP providers will be based on whether they are accredited by PPB. P&PT will submit a letter of good standing from PSK or KPA. They should be working in a PPB-licensed premise at the time of admission to training and be a registered pharmacist or an enrolled pharmaceutical technologist with the PPB and an up-to-date member of PSK or KPA.

## 3. Summary

### 3.1. Key achievements

Advocacy for the implementation of the policy that allowed P&PT to provide injectable contraceptives resulted in the MOH developing and rolling out an in-service FP training package and developing a pharmacy reporting platform within the national health information system.

At the pre-service level, advocacy resulted in revision of the PPB's core curriculum to include FP. The revision also incorporated other important aspects such as gender, supply chain, immunization, and leadership and governance. An initial group of five universities are at various stages of implementing FP training within their respective pharmacy departments given that the module has been included in the national preservice curriculum.

### 3.2. Challenges

AFP faced several challenges along the advocacy journey. Transitions of key decision-makers within the MOH meant that advocates had to continually bring the new leaders on board, which slowed the process. There were also many key stakeholders who advocates needed to get buy-in from to move together as a team. Even after amendment of the FP guidelines to allow P&PT to provide injectable contraceptives, dissemination of training, and issuance of a circular on pharmacy reporting platform, there were actors and cadres within the MOH who were unaware of these developments. Some of these assumed pharmacists were providing services illegally by offering injectable contraceptives. To allay fears, the MOH issued certificates to those trained in providing FP services, including injectable contraceptives. The media was also engaged to increase visibility of the policy change.

Introduction of a new pharmacy reporting platform to a cadre that rarely reported to the national health information system also meant that the Department of Monitoring, Evaluation and Informatics had to continually orient and provide support services to the newly trained P&PT.

### 3.3. Facilitators

Engaging the PSK—a professional organization representing pharmacists—as an advocacy ally accelerated buy-in by pharmacists and pharmacy departments in educational institutions. Given that policy implementation involves many processes that require resources, the establishment of technical and coordination committees that included implementing partners helped to leverage resources.

Engaging the PPB, which is highly revered, at the very onset of policy implementation helped to advance consensus and a harmonious working relationship, which were vital in achieving milestones. It also preempted formation of barriers that often emerge when critical stakeholders are not involved.

Overall leadership by the MOH's Department of Family Health in the development of the pharmacists' FP training package increased ownership by government and deepened understanding of the issues and content of the package by technical managers within the department. These managers played a key role in engaging with other departments and regulatory bodies to make a case for achieving other milestones during the policy implementation advocacy phase.

Proper timing on when to advocate for each implementation milestone increased the chances for actualizing desired outcomes. For example, engaging the PPB to review the pre-service core curriculum when there was a growing ideological shift to improve the training and pharmacy practice helped to advance the curriculum review.

### 3.4. Lessons learned

Based on our experience, the following are key considerations and lessons learned that can facilitate adoption of this task-sharing model in other similar contexts.

#### 3.4.1. Advocacy is a critical ingredient in translating best practices into policy and subsequent implementation

While the World Health Organization had issued guidelines that recommended task sharing in provision of injectable contraceptives by pharmacists, advocacy was needed to translate the global guidance into Kenya's policy. Because task sharing may not be easily adopted, due to the interests of the various cadres, advocacy with all players can be essential for buy-in.

#### 3.4.2. Early involvement of regulatory bodies in the policy change opens doors for implementation by different player

Regulatory bodies are mandated to set standards for the education, training, and practice of the various health cadres and to monitor the practices of health workers, to ensure that they comply with those standards. Given that P&PT were not allowed to provide injectable contraceptives as per their scope of practice, engagement of regulatory bodies, specifically the PPB, at the onset of this process was imperative to ensure expansion of services provided by this cadre.

#### 3.4.3. Engagement of professional bodies in policy change and implementation facilitates buy-in from providers

Almost all health cadres have professional associations that champion their members' interest. Regulatory changes that culminate into policies are not adequate for implementation. Policy changes that affect health cadres need to get buy-in from the members through their association. It is against this backdrop that the project engaged the PSK (for pharmacists) and KPA (for pharmaceutical technologists) for ownership of this process.

#### 3.4.4. Leadership and commitment by the ministry of health is crucial for successful roll-out

The MOH must lead and commit to implementing policy changes related to task sharing. In-service training of P&PT who were already providing services was led by the MOH, including quality assurance of service providers, linkage with the public sector for referrals, and reporting.

#### 3.4.5. Sustaining task-sharing advocacy gains requires institutionalization by academic institutions

To ensure the sustainability of the policy change, it was essential to engage academia in promoting pre-service training for students. Working with academic institutions that train P&PT led to the incorporation of a FP module into their curriculum. Incorporating FP-related content into P&PT's education was essential to improving and strengthening FP service delivery, as it allows students to develop a solid foundation of core knowledge and skills in FP. This has the potential to reduce the cost of in-service training.

## 4. Conclusion

Policy formulation does not necessarily translate to policy implementation. Advocacy is often needed to move policy to practice, especially when resources are required. Policy implementation also demands the incremental achievement of different milestones, hence the need for advocacy at every step along the way. Implementation of the policy that not only allows and but also supports P&PT to provide injectable contraception in Kenya has implications for other programs and other countries, especially in an era where there is a high unmet need for FP and health workers are under-resourced and over-burdened. For women and teenagers who might be hesitant to get contraception at medical facilities, the policy reform provides an alternative place for them to receive care. Additionally, it increases the private sector's contribution to mitigating commodities shortages.

Conversely, implementation of the policy that allows P&PT to provide injectable contraception in Kenya provides a reference and a way forward for allowing pharmacists to offer other primary health care services, such as immunization, injectable HIV prophylaxis, and other injection-oriented services not provided for in policy.

Private pharmacies can provide an alternative point of access for FP during public sector industrial strikes, disease outbreaks, and other public health emergencies, which can reduce missed opportunities for providing FP care. In addition, private pharmacists who source their own injectable contraceptives may reduce public health commodity expenditures in a context where injectables are the most popular method in Kenya and in sub-Saharan Africa, a move that promotes a total market approach.

Introducing FP training, including injectable contraceptives, during pre-service education is likely to reduce the cost of in-service training and contribute to sustainability of FP programs. Educational institutions can plough the cost of such training into the regular training, which can reduce the need for donor support to finance training—a common phenomenon for in-service training. Moreover, the data from the pharmacy reporting platform will eventually help with commodity forecasting and financing and can create an opportunity for other reporting areas that may be required of the pharmacy practice.

## Data Availability

The original contributions presented in the study are included in the article/Supplementary Material, further inquiries can be directed to the corresponding author.
